# Validation of the intolerance of uncertainty scale as a screening tool for perinatal anxiety

**DOI:** 10.1186/s12884-021-04296-1

**Published:** 2021-12-14

**Authors:** Melissa Furtado, Benicio N. Frey, Sheryl M. Green

**Affiliations:** 1 Department of Psychology, Neuroscience and Behaviour, McMaster University, 1280 Main Street West, Ontario, Hamilton L8S 4L8 Canada; 2 Women’s Health Concerns Clinic, St. Joseph’s Healthcare Hamilton, Level 1, 100 West 5th Street, Hamilton, Ontario L8N 3K7 Canada; 3 Department of Psychiatry and Behavioural Neurosciences, McMaster University, Administration B3, 100 West 5th Street, Hamilton, Ontario L8N 3K7 Canada; 4 Mood Disorders Program, St. Joseph’s Healthcare Hamilton, Level 1, 100 West 5th Street, Hamilton, Ontario L8N 3K7 Canada

**Keywords:** Anxiety, Perinatal, Pregnancy, Postpartum, Screening, Intolerance of uncertainty

## Abstract

**Background:**

To date, there is a significant lack of research validating clinical tools for early and accurate detection of anxiety disorders in perinatal populations. Intolerance of uncertainty was recently identified as a significant risk factor for postpartum anxiety symptoms and is a key trait of non-perinatal anxiety disorders. The present study aimed to validate the Intolerance of Uncertainty Scale (IUS) in a perinatal population and evaluate its use as a screening tool for anxiety disorders.

**Methods:**

Psychiatric diagnoses were assessed in a sample of perinatal women (*n* = 198), in addition to completing a self-report battery of questionnaires. Psychometric properties including internal consistency and convergent and discriminant validity were assessed. Determination of an optimal clinical cut-off score was measured through a ROC analysis in which the area under the curve, sensitivity, specificity, as well as positive and negative predictive values were calculated.

**Results:**

The IUS demonstrated excellent internal consistency (α = 0.95) and an optimal clinical cut-off score of 64 or greater was established, yielding a sensitivity of 89%. The IUS also demonstrated very good positive (79%) and negative (80%) predictive values.

**Conclusions:**

These findings suggest that the IUS represents a clinically useful screening tool to be used as an aid for the early and accurate detection of perinatal anxiety.

## Background

As many as 1 in 4 individuals globally are diagnosed with an anxiety disorder in their lifetime, in which women are twice as likely to be diagnosed compared to men [1]. Women account for approximately 65% of the 26.8 million disability adjusted life years (DALYs) [2, 3] and anxiety disorders are associated with substantial economic burden. Anxiety, together with depression, accounts for over $1 trillion per year in healthcare and lost productivity [4], with anxiety disorders in Canada alone costing the economy $17.3 billion per year [5].

Until recently, perinatal mental health research has focused on postpartum depression (PPD) which affects as many as 12% of women [6], however, there has been increasing awareness that the perinatal period is also associated with high risk for anxiety disorders. In fact, anxiety disorders during the perinatal period have been shown to be more prevalent than PPD, with rates between 15–24% [7]. Further, numerous negative effects are associated with perinatal anxiety disorders for both mothers and their infants. For instance, in addition to the distress and impairment associated with an anxiety disorder, anxiety during pregnancy has been associated with increased obstetric complications such as preterm birth and lower birth weight [8–11]. Women with perinatal anxiety utilize greater health resources, such as making more frequent visits to their obstetrician [12, 13] and have increased absenteeism from work because of their anxiety [8]. These negative effects also impact the mother-infant bond, such that women are more likely to report reduced perceived bonding with their infant [14]. Infants of mothers with perinatal anxiety experience greater cognitive and attention difficulties [15, 16] and are more likely to experience their own anxiety later in life [17–19]. Despite the high prevalence rates of perinatal anxiety disorders and associated negative effects, less than 15% of women receive appropriate treatment [20], often due to difficulties in timely and accurate symptom detection.

A lifetime history of mood and/or anxiety disorders are among the strongest predictors of perinatal anxiety [13, 21, 22]. Sociodemographic risk factors on the other hand, such as maternal age, parity, and education level, have demonstrated inconsistent findings in the literature [23–26]. More recently, a key trait of anxiety disorders known as intolerance of uncertainty, was identified as a significant risk factor for postpartum anxiety worsening in women with pre-existing anxiety disorders [27]. Intolerance of uncertainty results from negative beliefs about uncertainty and its potential negative implications [28]. Intolerance of uncertainty is any type of emotional, cognitive, and/or behavioural response to uncertainty which biases information processing, resulting in perceived negative implications [29]. It is a common characteristic across anxiety disorders [30–33] and is positively correlated with worry symptoms [34–36]. Further, intolerance of uncertainty has been demonstrated as a significant predictor and mediator of treatment response to cognitive behavioural therapy in non-perinatal populations [37].

To date, there is a paucity of research validating clinical tools for anxiety disorders in perinatal populations. Among self-report screening tools for perinatal anxiety are the Edinburgh Postnatal Depression Scale (EPDS), the Generalized Anxiety Disorder 7-Item Scale (GAD-7), the Perinatal Anxiety Screening Scale (PASS) and the Anxiety Disorder—13 Scale (AD-13). Although the EPDS is a well-validated screening tool for PPD [38, 39] and has been used as a multidimensional tool (EPDS-3A) to screen for perinatal anxiety, it is associated with a high rate of false positives [40] and is not recommended for widespread use as a screening tool for perinatal anxiety disorders [41]. The GAD-7 is one of the most commonly used self-report questionnaires in assessing anxiety symptom severity in the general population [42] and has been validated for use in the perinatal period [43]. Although the GAD-7, and the abbreviated GAD-2, perform slightly better than the EPDS-3A in detecting symptoms of GAD in perinatal women, they too, have not been recommended as a perinatal anxiety disorder screening tool for widespread use [41]. Unlike the EPDS and GAD-7, the PASS [44] was specifically designed to screen for a broad range of anxiety symptoms during pregnancy and the postpartum. Utilizing the validated clinical cut-off score of 26 or greater, the PASS has demonstrated fair accuracy (68%) in identifying perinatal women with an anxiety diagnosis compared to the EPDS-3A and GAD-7 [44]. Psychiatric diagnoses in this study, however, were not confirmed through the use of a standardized psychiatric interview (e.g., MINI, SCID), which may have limited the accuracy of diagnoses. The AD-13, which identifies core symptoms of anxiety disorders, has been found to better perform at accurately identifying anxiety disorders during the perinatal period [41]. Of note however, the AD-13 includes questions assessing Obsessive-Compulsive Disorder and Posttraumatic Stress Disorder, which are not currently considered anxiety disorders, as per the Diagnostic and Statistical Manual of Mental Disorders [41].

Given that intolerance of uncertainty has been well-documented as a risk factor for anxiety disorders in the general population and more recently in a perinatal population and shown to be a significant predictor of treatment response, it would be of great value to validate the IUS as a clinical tool for perinatal anxiety disorder screening. The 27-item Intolerance of Uncertainty Scale (IUS) is among the most commonly utilized and validated self-report questionnaire assessing intolerance of uncertainty. Therefore, the objective of the present study was to validate the psychometric properties of the IUS as a screening tool for clinical anxiety disorders in pregnant and postpartum women. Further, as clinical cut-off scores can be especially beneficial in screening for psychiatric disorders, we examined whether an optimal cut-off score could be achieved for detection of an anxiety disorder during the perinatal period. We hypothesized that the IUS would display high validity and reliability in detecting the presence of an anxiety disorder during the perinatal period. We further hypothesized that a cut-off with high sensitivity and predictive value would be determined in predicting the presence of an anxiety disorder during the perinatal period.

## Methods

### Participants and Procedures

Pregnant (≥14 weeks gestation) and postpartum (≤6 months) women, 18 years or older were enrolled in the present study. As the first trimester of pregnancy is associated with highest medical risk and in turn, understandable levels of anxiety, participants were recruited beginning in their second trimester of pregnancy to better identify those who would be more likely to experience anxiety symptoms which would persist. The time interval used to determine the postpartum period differs among studies and may be as high as 12 months postpartum [45]. Given that the prevalence rates of anxiety disorders are highest by 6 months postpartum [7, 26, 46–48], the present study utilized these criteria to define the postpartum period. Participants were recruited from the Women’s Health Concerns Clinic at St. Joseph’s Healthcare Hamilton, an outpatient mental healthcare clinic, prior to receiving psychological treatment [49]. Participants were also recruited through advertising in midwifery, physician clinics, and online (e.g., Kijiji) throughout the Greater Toronto and Hamilton area, between January 2020 to February 2021. Once eligibility was determined, participants completed a research study visit in which psychiatric diagnoses were assessed by the Mini International Neuropsychiatric Interview (MINI), version 7.0.2 for the Diagnostic and Statistical Manual of Mental Disorders, Fifth Edition (DSM-5). Upon completion of the study visit, participants were divided into two cohorts for analyses: those with a DSM-5 primary anxiety disorder and those without. Co-morbid secondary conditions were accepted for both cohorts so that the results of the study resemble what is observed in the real-world.

In addition to the MINI, participants completed a battery of self-report questionnaires *(see Study Measures below)* assessing clinical symptom severity of anxiety, worry, mood, and emotion regulation. To assess test-retest reliability, participants who agreed to participate in a second study visit, repeated the self-report questionnaire battery two-weeks after completion of their initial study visit. Study data was electronically stored and managed with the Research Electronic Data Capture (REDCap) system, which is a secure web-based application designed for research data collection [50]. This study was conducted in accordance with the Declaration of Helsinki and was approved by the Hamilton Integrated Research Ethics Board. All participants provided written informed consent.

### Study Measures

A brief demographics questionnaire was included in the battery of self-report questionnaires administered to participants. The demographics questionnaire included questions pertaining to the participant’s age, perinatal status, ethnicity, marital status, parity, education level, and medical history (e.g., medication use).

The *Intolerance of Uncertainty Scale (IUS)* is a 27-item self-administered questionnaire assessing one’s beliefs and reactions to uncertain events, ambiguity, and the future [29, 31]. Items are scored on a 5-point Likert scale, ranging from 1 (not at all characteristic of me) to 5 (entirely characteristic of me), with total possible scores of 27 to 135. Although initial validation studies have established a multifactorial structure, scores are most often reported as a total scale score. The IUS has demonstrated excellent internal consistency (α = 0.91–0.95) and good test-retest reliability (*r* = 0.78) in general (non-perinatal) populations.

The *Generalized Anxiety Disorder 7-Item Scale (GAD-7)* is a 7-item self-report questionnaire assessing anxiety symptom severity for the previous two-week period [42]. Items on the GAD-7 are measured on a 4-point Likert scale ranging from 0 (not at all) to 3 (nearly every day). The GAD-7 has good sensitivity (89%) and specificity (82%) in detecting a clinical diagnosis of GAD, when a cut-off score of 10 or higher is utilized. The GAD-7 has also been validated in a perinatal population, yielding adequate sensitivity (61.3%) and specificity (72.7%) with an optimal cut-off score of 13 or higher [43].

The *Edinburgh Postnatal Depression Scale (EPDS)* is a 10-item self-report questionnaire assessing perinatal depression [38]. Items are scored on a 4-point Likert scale, with higher scores indicating greater depressive symptom severity. The EPDS has demonstrated good sensitivity and specificity at 86 and 78%, respectively, for a diagnosis of Major Depressive Disorder when a clinical cut-off score of 10 or higher is utilized. Recent studies, however, have demonstrated a cut-off score of 13 or higher for the detection of postpartum depression specifically [51]. The EPDS has also been used to assess postpartum anxiety, with 3 of the 10 included questions specifically probing into anxiety symptoms [40].

The *Penn State Worry Questionnaire (PSWQ)* is a 16-item self-administered questionnaire assessing worry symptoms [52]. Items are scored on a 5-point Likert scale, ranging from 1 (not at all typical of me) to 5 (very typical of me), with scores at or above 65 representing a clinically significant level of worry [53]. The PSWQ has been considered a gold-standard for assessing worry and has demonstrated excellent psychometric properties in both perinatal and non-perinatal population [54–56].

The *State-Trait Inventory for Cognitive and Somatic Anxiety (STICSA)* consists of two 21-item self-report subscales assessing state and trait anxiety [57]. The “state” subscale assesses the individuals current, *at this very moment*, anxiety, while the “trait” subscale refers to how individuals feel *in general.* For the purposes of the present study, the trait subscale was utilized. Items are scored on a 4-point Likert scale ranging from 1 (not at all) to 4 (very much so). The STICSA can be scored to assess cognitive anxiety symptoms (e.g., rumination, intrusive thoughts) and somatic anxiety symptoms (e.g., dizziness, sweating, heart racing) separately. The STICSA has demonstrated excellent validity and reliability [58]. To detect the presence of a clinical anxiety disorder, a cut-off score of 43 or higher has been suggested [59].

The *Difficulties in Emotion Regulation Scale (DERS)* is a 36-item self-report questionnaire assessing six dimensions of emotion regulation: non-acceptance, goals, impulse, awareness, strategies, and clarity [60]. Items on scored on a 5-point Likert scale ranging from 1 (almost never, 0–10%) to 5 (almost always, 91–100%), with higher scores indicating greater difficulties in regulating one’s emotions. The DERS has demonstrated good internal consistency and test-retest reliability [61].

### Statistical Analyses

Independent samples t-tests were performed to compare continuous variables (e.g., age) between participants with and without a primary anxiety disorder. Chi-square tests were utilized to assess group differences for categorical data (e.g., parity). Reliability of the IUS was assessed by measuring internal consistency using Cronbach’s alpha and with test-retest reliability. To assess convergent and discriminant validity, correlations with well-established measures of worry, anxiety, depression, and emotion regulation were measured. To assess criterion validity, a receiver operating characteristic (ROC) analysis was utilized to estimate the sensitivity and specificity pairings, the area under the curve (AUC) and 95% confidence interval (CI) for a range of cut-off scores for the IUS. The AUC was used to determine the screening accuracy of the IUS in predicting a primary anxiety disorder during the perinatal period. The optimal clinical cut-off score of the IUS was set by the largest Youden Index (YI), which is derived from the sum of sensitivity and specificity minus one. Based upon the optimal clinical cut-off of the IUS, as determined by the YI, sensitivity, specificity, positive (PPV) and negative (NPV) predictive values were calculated. To confirm the accuracy and specificity of the IUS as an anxiety disorder screening tool during the perinatal period, a ROC analysis, calculation of an optimal cut-off score and associated sensitivity, specificity, PPV and NPV were calculated to also assess the use of the IUS as a screening tool for primary and/or secondary depressive disorders (e.g., Major Depressive Disorder, Persistent Depressive Disorder). The level of statistical significance was set at a *p*-value <0.05. All statistical analyses were performed with IBM SPSS Statistics 23 [62].

## Results

Pregnant (*n* = 92) and postpartum (*n* = 106) women meeting all inclusion/exclusion criteria were enrolled in the present study, for a total of 198 participants. Participants ranged in age from 19 to 44 years, with a mean age of 31.8 years (SD = 4.37). In assessing current psychiatric diagnoses, as per the MINI for DSM-5, 118 participants met criteria for a primary anxiety disorder and 80 participants did not. The most common primary anxiety disorder was GAD (89.8%), followed by Social Anxiety Disorder (5.9%) and Panic Disorder (4.2%). Of the 80 participants without a primary anxiety disorder, 51.2% did not meet criteria for any lifetime psychiatric disorders, 22.5% had past Major Depressive Disorder (MDD), while 5% had current MDD. Baseline characteristics for participants are outlined in Table 1.

**Table 1:** Baseline demographics and characteristics (*n* = 198)

	Primary AD (***n*** = 118)	Control (***n*** = 80)	***P***-value
**Mean age (SD)**	31.1 (4.57)	32.8 (3.85)	0.07
**Perinatal Status**			
**Pregnant**	47 (39.8%)	45 (56.2%)	0.02
**Postpartum**	71 (60.2%)	35 (43.8%)	
**Ethnicity**			
**Caucasian**	98 (83.1%)	60 (75%)	0.05
**Black**	2 (1.7%)	0 (0%)	
**First Nations**	2 (1.7%)	0 (0%)	
**Latino/Hispanic**	1 (0.9%)	2 (2.5%)	
**Middle Eastern**	1 (0.9%)	3 (3.8%)	
**South Asian**^**a**^	0 (0%)	3 (3.8%)	
**East Asian**^**b**^	0 (0%)	3 (3.8%)	
**Asian/Pacific Islander**	4 (3.4%)	1 (1.2%)	
**Other**	10 (8.5%)	8 (10%)	
**Marital Status**			
**Single**	6 (5.1%)	1 (1.2%)	0.14
**Married/Common-law**	111 (94.1%)	76 (95%)	
**Divorced**	1 (0.8%)	3 (3.8%)	
**Parity**			
**Primigravida**	81 (68.6%)	47 (58.8%)	0.15
**Multigravida**	37 (31.4%)	33 (41.2%)	
**Education**			
**≤ High school**	12 (10.2%)	9 (11.2%)	0.002
**College/University**^**c**^	87 (73.7%)	41 (51.2%)	
**Postgraduate (e.g., MD, PhD)**^**d**^	19 (16.1%)	30 (37.5%)	

Post-hoc analyses for chi-square tests revealed statistically significant differences between the primary AD and control groups in ethnicity (^a^South Asian, ^b^East Asian) and education level (^c^College/University and ^d^Postgraduate)

### Reliability and Validity

The IUS demonstrated excellent internal consistency (α = 0.95) in the present sample. To assess test-retest reliability, a sample of participants (*n* = 35) repeated the study measures two weeks after their baseline assessment. In test-retest analysis, the correlation between baseline and follow-up IUS scores was excellent (*r* = 0.91). To assess convergent and discriminant validity, Pearson correlations between IUS scores and included self-report measures was examined. Further convergent validity was demonstrated between the IUS and PSWQ (*r* = 0.75, *p* < 0.001), GAD-7 (*r* = 0.73, *p* < 0.001), STICSA total scale (*r* = 0.74, *p* < 0.001) and cognitive subscale (*r* = 0.79, *p* < 0.001), and DERS (*r* = 0.74, *p* < 0.001). Although all correlations were statistically significant, the strength of correlations between the IUS and EPDS depression subscale (*r* = 0.55, *p* < 0.001) and STICSA somatic subscale (*r* = 0.53, *p* < 0.001) were considered moderate demonstrating discriminant validity.

### Diagnostic Accuracy

**Fig. 1:**
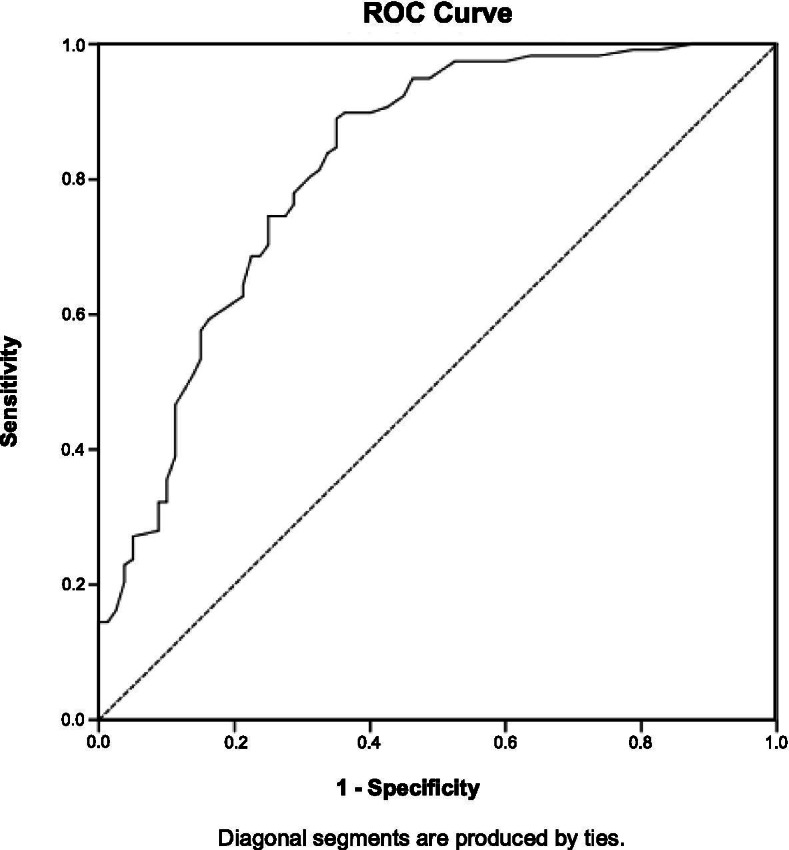
Receiver operating characteristic (ROC) curve for the IUS to detect primary anxiety disorders during the perinatal period

The ROC curve for the IUS is illustrated in Fig. 1. The AUC was calculated to examine the performance of the IUS as a screening tool in detecting the presence of an anxiety disorder in perinatal women. Accuracy was interpreted as having low (AUC = 0.50 to 0.70), acceptable (AUC = 0.70 to 0.80), excellent (AUC = 0.80 to 0.90), and outstanding (AUC = 0.90 or greater) discrimination [63]. The AUC of the IUS was calculated as 0.82 (95% CI: 0.76–0.88), indicating that the IUS had excellent screening accuracy for primary anxiety disorders among perinatal women. When maximizing the Youden Index (YI = 0.54), an optimal clinical cut-off score of 64 was found. Sensitivity and specificity at the optimal clinical cut-off score of 64 or greater was 89 and 65%, respectively. Further, the IUS demonstrated very good PPV (79%) and NPV (80%).

In assessing the use of the IUS as a depression screening tool during the perinatal period, the AUC was calculated as 0.59 (95% CI: 0.51–0.69), indicating low discrimination. When maximizing the Youden Index (YI = 0.24, interpreted as low), an optimal clinical cut-off score of 76 was found. Sensitivity and specificity at the optimal clinical cut-off score of 76 or greater was 69 and 55%, respectively. Further, the IUS demonstrated good NPV (87%), however very poor PPV (27%) in predicting the presence of a depressive disorder during the perinatal period.

## Discussion

Anxiety disorders during the perinatal period are highly prevalent and are associated with significant burden and negative outcomes for both mother and child. Unlike other disorders such as perinatal depression, there are very few screening tools which have been validated for use in perinatal anxiety. Further, the screening tools which have been validated in a perinatal population have demonstrated high false positive rates and are not recommended for widespread use in screening of perinatal anxiety disorders.

This was the first study to specifically examine the use of the IUS as a screening tool for perinatal anxiety disorders. Previous research has demonstrated the importance of intolerance of uncertainty in anxiety disorders both in the general population [30–36], and more recently in the perinatal population [27] in which higher scores were associated with postpartum anxiety worsening. Overall, the current results support the use of the IUS as a potential screening tool for perinatal anxiety disorders. The 27-item IUS demonstrated excellent internal consistency and test-retest reliability among our sample of pregnant and postpartum women. The IUS was also positively correlated with measures of anxiety and worry, demonstrating convergent validity. Of note, the IUS was correlated with STICSA total scores and cognitive subscale scores, but less so with somatic subscale scores. Given that intolerance of uncertainty is defined as a cognitive bias impacting the way one perceives, interprets, and responds to uncertain events, it is understandable as to why the IUS would not be strongly correlated to somatic anxiety symptoms (e.g., heart racing), which demonstrates discriminant validity. Similarly, the IUS demonstrated convergent validity with the DERS, which is a measure of emotion dysregulation. Individuals with anxiety disorders exhibit strong emotional reactions and often have difficulties interpreting their emotions, which can further exacerbate their worry and use of maladaptive coping behaviours [64–68]. Further, intolerance of uncertainty has been known to contribute to worry via negative problem orientation. Individuals high in intolerance of uncertainty tend to exhibit pessimistic views towards any potential problem or uncertain situations, perceiving them as threats and, in turn, doubting their abilities to cope with or resolve them if needed [69]. Due to this negative problem orientation, these individuals will often avoid any uncertain situation reinforcing their cognitive biases and producing anxiety and emotional distress [70, 71]. Recent research has also revealed that emotion dysregulation is significantly associated with anxiety symptoms during pregnancy [72] and is a significant mediator of the relationship between intolerance of uncertainty and worry in a non-perinatal population [69]. As emotional states are intrinsically linked to uncertainty, and given the relationship between emotion dysregulation and anxiety, it is understandable as to why convergent validity was exhibited between the IUS and DERS.

The present study also demonstrated discriminant validity between the IUS and EPDS, particularly the EPDS depression subscale which removes the three anxiety items. Although the correlation between these measures were significant, the relationship was moderate therefore supporting discriminant validity. Perinatal anxiety and depressive disorders are highly comorbid [73–76] and overlapping symptomatology may even hinder accurate symptom detection [7]. Limited research has also demonstrated the association between intolerance of uncertainty and depressive symptomatology, suggesting that high intolerance of uncertainty may be a risk factor for the development of depression [77, 78]. The findings however have been robust in nature and require further investigation. As intolerance of uncertainty and depression are associated, it is reasonable as to why the IUS, which assesses a key trait in anxiety disorders, would be significantly correlated with the EPDS. Nevertheless, the correlation was to a moderate degree, as the items on the EPDS assess distinctive depressive symptoms such as anhedonia, hopelessness, and self-injurious behaviour, which are not always exhibited in anxiety disorders. Further, in assessing the use of the IUS as a screening tool for depressive disorders during the perinatal period, the IUS demonstrated poor screening accuracy (AUC = 0.59). These results suggest that although intolerance of uncertainty has been found to be associated with depression, it is not a good screening tool to detect the presence of depressive disorders during the perinatal period. Instead, the results suggest the specificity of the IUS in screening for anxiety disorders during the perinatal period and other validated screening tools such as the EPDS are suggested for depressive disorder screening.

The accuracy of the IUS as a screening tool, as assessed by the AUC, was interpreted as excellent at 0.82. The AUC of the IUS in this study is greater than those measures (e.g., EPDS-3A, GAD-7) which are commonly used as screening tools for perinatal anxiety [41, 43, 79]. Sensitivity of the IUS in detecting a primary perinatal anxiety disorder was excellent, while specificity was fair. When validating a measure to be utilized in clinical populations as a screening tool, positive and negative predictive values are considered more relevant than sensitivity and specificity [80]. Higher NPV and PPV values are recommended to demonstrate accuracy in screening detection [41, 80]. In the present study, the NPV was calculated as 80%, which is the probability that individuals who score below the optimal clinical cut-off score of 64 on the IUS, truly do not have a primary anxiety disorder. An NPV of ≥80% suggests that the screening tool being utilized (i.e., IUS) is comparable to what is considered the gold standard for diagnoses [81], such as a structured clinical interview for psychiatric disorders. Similarly, a higher PPV is recommended for clinical screening tools, as it is interpreted at the true positive rate. In this study, the PPV of the IUS was 79%, suggesting that 79% of those individuals scoring ≥64 on the IUS did have a primary anxiety disorder. As NPV and PPV are more relevant in clinical screening, the IUS demonstrated excellent anxiety disorder screening abilities when a cut-off score of ≥64 is utilized to detect the presence of a primary anxiety disorder during the perinatal period.

### Limitations

Although the present study was successful at revealing the use of the IUS as a screening tool for anxiety disorders during the perinatal period, there are some limitations to consider. Despite the psychometric properties of the IUS being established in non-perinatal populations, there is not an accepted clinical cut-off for anxiety disorders in those populations. Although previous research has revealed the association between perinatal anxiety and intolerance of uncertainty, which is consistent with the non-perinatal intolerance of uncertainty research, we are unable to compare whether perinatal intolerance of uncertainty levels are comparable to non-perinatal levels. The lack of a comparison group, specifically non-perinatal participants with and without anxiety disorders, is therefore a limitation of the present study. Positive and negative predictive values are highly dependent on the prevalence of a condition in the tested sample. Specifically, as the prevalence of the condition (e.g., anxiety disorder) increases, so does the positive predictive value, while the negative predictive value decreases. Although the included sample consisted of perinatal women across various settings (e.g., clinical settings, community), some participants were recruited from mental healthcare clinics and therefore the prevalence of anxiety disorders may have already been greater, which may have impacted the positive and negative predictive values. Replication studies in the future could recruit participants solely from the community, and not mental healthcare settings. Generalizability in relation to sociodemographic variables is limited, as the majority of the sample was primarily Caucasian and highly educated. With respect to the anxiety disorder sample, the majority of participants within this group had GAD as their primary anxiety disorder. Although this is consistent with the current literature in which GAD is considered the most prevalent perinatal anxiety disorder [82], we were unable to determine if there are any differences in intolerance of uncertainty between anxiety disorders. Given that intolerance of uncertainty is considered a key trait across all anxiety disorders, however, we would hypothesize that future studies separating anxiety disorders during the perinatal period would yield similar results.

## Conclusion

The present study was the first to investigate the psychometric properties of the IUS for use in a perinatal population. The findings demonstrate that the IUS represents a clinically meaningful screening tool to be used in perinatal populations to aid in the early and accurate detection of anxiety disorders. Higher scores on the IUS significantly predicted the presence of a primary anxiety disorder and established an optimal clinical cut-off score of ≥64. Pregnant and postpartum women who often go undiagnosed and, in turn, untreated for anxiety disorders face both short- and long-term consequences for themselves and their children. Screening measures can significantly improve symptom detection and reduce, or even prevent, these unwanted negative outcomes. Routine administration of the IUS across maternity and perinatal care settings (e.g., midwifery clinics, obstetrics and gynecology) can serve as a valuable screening tool to improve early detection of anxiety symptoms during pregnancy and the postpartum. Although the IUS consists of 27 items, the item statements are relatively concise and relatively brief to administer so as not to over burden the patient. Given the importance of intolerance of uncertainty in anxiety disorders and in predicting treatment response, the IUS is an easily administered self-report questionnaire which may provide useful information for clinicians in early and accurate symptom detection and diagnoses.

## Data Availability

The dataset for the current study is available from the corresponding author upon reasonable request.

## Abbreviations

AUC: Area under the curve
CI: Confidence interval
DALYs: Disability adjusted life years
DERS: Difficulties in Emotion Regulation Scale
DSM-5: Diagnostic and Statistical Manual of Mental Disorders, Fifth Edition
EPDS: Edinburgh Postnatal Depression Scale
GAD: Generalized Anxiety Disorder
GAD-7: Generalized Anxiety Disorder 7-Item Scale
IUS: Intolerance of Uncertainty Scale
MDD: Major Depressive Disorder
MINI: Mini International Neuropsychiatric Interview
NPV: Negative predictive value
PASS: Perinatal Anxiety Screening Scale
PPD: Postpartum depression
PPV: Positive predictive value
PSWQ: Penn State Worry Questionnaire
ROC: Receiver operating characteristic
REDCap: Research electronic data capture
SCID: Structured Clinical Interview for the DSM-5
STICSA: State-Trait Inventory for Cognitive and Somatic Anxiety
SD: Standard deviation
YI: Youden’s Index
